# Esophageal Intramural Pseudodiverticulosis and Concomitant Eosinophilic Esophagitis: A Case Series

**DOI:** 10.1155/2016/1761874

**Published:** 2016-08-28

**Authors:** Michael A. Scaffidi, Ankit Garg, Brandon Ro, Christopher Wang, Tony T. C. Yang, Ian S. Plener, Andrea Grin, Errol Colak, Samir C. Grover

**Affiliations:** ^1^Division of Gastroenterology, St. Michael's Hospital, Toronto, ON, Canada; ^2^Laboratory Medicine and Pathobiology, St. Michael's Hospital, Toronto, ON, Canada; ^3^Department of Medical Imaging, St. Michael's Hospital, Toronto, ON, Canada

## Abstract

*Background*. Esophageal intramural pseudodiverticulosis (EIPD) is an idiopathic benign chronic disease characterized by flask-like outpouchings of the esophageal wall. It is unknown whether there is a genuine association between EIPD and eosinophilic esophagitis (EoE).* Aims*. To investigate a possible relationship between EIPD and EoE.* Methods*. Patients with radiographic or endoscopic evidence of pseudodiverticulosis were identified from the database at a single academic center. Cases were analyzed in three areas: clinical information, endoscopic findings, and course.* Results*. Sixteen cases of esophageal pseudodiverticulosis were identified. Five patients had histologic evidence of eosinophilic esophagitis. Patients with EoE had pseudodiverticula in the mid-to-distal esophagus while those with EIPD had pseudodiverticula predominantly in the proximal esophagus (*p* < 0.001). EoE with pseudodiverticulosis occurred in younger patients (*p* < 0.019). Food bolus obstructions were more common in patients with EoE and pseudodiverticulosis than in EIPD (*p* < 0.034).* Conclusions*. This is the first case series supporting a potential association between EoE and pseudodiverticulosis. We also identify characteristic features of pseudodiverticulosis that may raise clinical suspicion of underlying eosinophilic esophagitis.

## 1. Introduction

Esophageal intramural pseudodiverticulosis (EIPD) is an idiopathic benign disease characterized by flask-like outpouchings of the esophageal wall distinguished by dilatation and inflammation of excretory ducts within the submucosal esophageal glands [1]. It is a rare condition with fewer than 250 cases reported [2]. Symptoms include progressive dysphagia or odynophagia and rarely gastrointestinal bleeding. The diagnosis is typically made by either esophagoscopy or barium esophagography. Proposed risk factors include alcohol and tobacco use [1] and potential associated comorbidities include gastroesophageal reflux, esophageal candidiasis, diabetes mellitus, chronic liver disease, and malnutrition [3].

There have been three case reports of concomitant eosinophilic esophagitis (EoE) with pseudodiverticulosis [4]. While both conditions present with dysphagia and result in esophageal mucosal change and stricturing [1, 5], the pathologic hallmark of EoE is mucosal eosinophilia, in contrast to the alteration of the submucosal esophageal glands in EIPD [6, 7]. Given the paucity of data, it is unknown whether there is a genuine association between EIPD and EoE. This case series evaluates the risk factors, medical comorbidities, and clinical and investigative findings among patients with esophageal pseudodiverticulosis, in order to evaluate a possible relationship between EIPD and EoE.

## 2. Methods

The radiographic and endoscopic patient databases were searched for records of patients with findings of esophageal pseudodiverticulosis between May 2000 and October 2015, at St. Michael's Hospital, a gastroenterology tertiary care center of the University of Toronto. Patients with either radiographic or endoscopic evidence of pseudodiverticulosis with a minimum of one year of follow-up after diagnosis were identified. All patients who present for diagnostic imaging are captured into the radiologic database. The endoscopic database is populated by gastroenterologists electively at the time of endoscopic procedures. Both databases are keyword searchable. Keywords in the search were “pseudodiverticulosis”, “esophageal intramural pseudodiverticulosis”, and “eosinophilic esophagitis”. Each case was individually reviewed to determine whether it met the following inclusion criteria: (1) radiographic and/or endoscopic diagnosis of esophageal pseudodiverticulosis; (2) minimum of two follow-up clinic visits with a gastroenterologist after the initial diagnosis; and (3) minimum of one follow-up esophagogastroduodenoscopy (EGD) after the initial diagnosis. We excluded any patients if they were under 18 years of age.

A diagnosis of pseudodiverticulosis was made on the basis of endoscopic findings using standard video endoscopes or radiological findings (on barium swallow) of pseudodiverticulosis. A diagnosis of eosinophilic esophagitis was made on the basis of (1) endoscopic evidence of EoE and (2) greater than 15 eosinophils per high power field from midesophageal biopsies. Location and distribution of pseudodiverticula in the esophagus were identified as proximal, mid-to-distal, or diffuse. The location of pseudodiverticulosis was extracted as documented on the endoscopy or radiology report.

Cases were analyzed in three areas: background patient information (demographics, medical history); investigative findings (from endoscopy, radiology, pathology, and cytology); and clinical course. Quantitative results are represented as mean values ± standard deviation (SD) or as median values with interquartile range (IQR). Categorical results are given as count and percentage values. Mann-Whitney *U* tests and Fisher exact tests were used to determine if there were differences in risk factors between patients with EIPD and EoE with pseudodiverticulosis. Exact probabilities were used to calculate *p* values. Two-sided alpha of 0.05 was used for all tests.

## 3. Results

A total of sixteen cases with findings of pseudodiverticulosis of the esophagus were identified. Demographics, comorbidities, clinical presentation, and endoscopic and histologic findings for all patients are summarized in Table 1. The mean age of diagnosis was 50.8 years. Fourteen of the sixteen patients had esophageal biopsies taken; the indication for biopsies varied. Five patients were also found to have a clinical diagnosis and histologic evidence of eosinophilic esophagitis (>15 eosinophils per high power field). Subgroup analyses comparing risk factors for EoE with pseudodiverticulosis and patients with EIPD are also listed in Table 1. Eosinophilic esophagitis with pseudodiverticulosis occurred in a younger patient population compared to patients with EIPD (*p* < 0.019). There was a significant difference between both groups with respect to anatomical localization of pseudodiverticula (*p* < 0.001). Specifically, those with EoE and pseudodiverticulosis had pseudodiverticula in the mid-to-distal esophagus while those with EIPD had pseudodiverticula predominantly in the proximal esophagus. There were significantly more food bolus obstructions found in the patients with EoE with pseudodiverticulosis as compared to the EIPD subgroup (*p* < 0.034). Finally, patients with EoE and pseudodiverticulosis were more likely to have allergies, including asthma or atopic dermatitis (*p* < 0.034).

The clinical course of all patients, including therapeutic interventions and outcomes, is listed in Table 2. Medical therapies were directed towards the underlying disease process. Patients with EoE were treated with a six-food elimination diet (2/5) or topical steroids (4/5). For EIPD, if GERD was present, patients were treated with proton-pump inhibitors (8/11), and if* Candida* was present, they were treated with antifungals (2/11). The mean total duration of follow-up was 44.4 months. No patients required surgical intervention or parenteral nutrition. One patient with EIPD died of non-small-cell lung cancer. Esophageal dilatation was an endoscopic modality used in treatment for both groups.

## 4. Discussion

Our case series is the first to offer evidence in support of a relationship between EoE and esophageal pseudodiverticulosis. Five patients with pseudodiverticulosis had endoscopic findings suggestive of EoE and met the histologic criteria for diagnosis (greater than 15 eosinophils per high power field) [7]. Concomitant presentation of esophageal pseudodiverticulosis and EoE has only been discussed in three case reports [4].

We found that the location of pseudodiverticulosis within the esophagus was different between the two patient subgroups. Patients with EoE tend to have pseudodiverticulosis within the mid-to-distal esophagus, while patients without EoE had pseudodiverticula primarily in the proximal esophagus. This finding is commensurate with three other case reports, which found that patients with EoE have segmental pseudodiverticulosis confined to the mid-to-distal esophagus [4].

In addition, EoE with pseudodiverticulosis occurred in younger patients than those with pseudodiverticulosis alone. The literature supports this claim, as the mean age of patients with EoE is approximately 33 years [8], while the mean age for patients with EIPD ranges from 58 to 62 years in recent studies [1, 5]. Moreover, compared to patients with EIPD, we found that patients with EoE and pseudodiverticulosis had significantly more food bolus obstructions, a known clinical presentation of stricturing disease caused by EoE [9]. As expected given the disease associations of EoE, allergies, including related conditions, such as asthma and atopic dermatitis, were more commonly seen in patients with EoE and pseudodiverticulosis than those with EIPD [8]. Finally, while alcohol, GERD, and diabetes mellitus are thought to be risk factors for EIPD [1], we did not see any differences between patients with EIPD and with EoE and pseudodiverticulosis for these factors.

Although our findings suggest a relationship between EoE and pseudodiverticulosis, the exact mechanism underlying this association is unknown. Pseudodiverticula are dilated excretory ducts of the esophageal submucosal gland [10]. Suggested mechanisms for formation of pseudodiverticula are (1) chronic inflammation; (2) debris deposition in the excretory ducts of the submucosal glands (which cause either obstruction or expansion of the glands); or (3) increased intraluminal pressure proximal to a luminal obstruction [6, 11, 12]. EoE can lead to all three of these phenomena,,the first as it is a chronic inflammatory condition, the second through recurrent food bolus obstruction, and the third due to the development of strictures which is common in EoE [8].

While three isolated cases of EoE and pseudodiverticulosis have been documented in the literature, the authors of these reports suggest this association is rare. However, our case series illustrates that this relationship may in fact be more common than previously thought given its manifestation in five of our sixteen patients. This may be explained by the fact that EoE has only more recently been recognized clinically as a disease entity [8].

Our findings must be placed in the context of several limitations. First, the lack of established guidelines for EIPD may impact clinicians' ability to make this diagnosis. Moreover, it is possible that cases of pseudodiverticulosis may have been missed on endoscopy, as pseudodiverticula are characteristically small. As a result of these two limitations, our data may be underrepresenting the association between EIPD and other conditions. Conversely, if many cases of pseudodiverticulosis were missed, it is likely that the association between pseudodiverticulosis and EoE would be overestimated. In addition, we were limited to data only available in the patients' medical records given that this was a retrospective study. For example, we did not have access to esophageal manometry studies for these patients. As such, further links between EIPD and EoE and other eosinophilia-related conditions could not be explored. Finally, although we demonstrate an association between EoE and pseudodiverticulosis, we were unable to establish the temporality of this relationship due to a paucity of data.

In light of our limitations, we make several conclusions relevant to clinical practice. We demonstrate that the association between EoE and pseudodiverticulosis may be more common than previously appreciated. Furthermore, we have identified characteristic features of pseudodiverticulosis that may raise clinical suspicion of underlying EoE. Mainly, these patients tend to have pseudodiverticulosis in the mid-to-distal esophagus. As well, these patients are typically younger, have a higher frequency of food bolus obstructions, and have the typical disease associations of EoE, atopy, asthma, and allergy. Further research characterizing the relationship between EoE and pseudodiverticulosis is required. Patients who have EoE with pseudodiverticulosis should be followed in the long term to evaluate the significance on the clinical course and outcome.

## Figures and Tables

**Table 1:** Demographics, comorbidities, clinical presentation, and endoscopic and histologic findings for all patients, including the subgroup analysis of patients with EIPD and with EoE and pseudodiverticulosis.

Characteristics	All patients (*n* = 16)	Patients with EIPD (*n* = 11)	Patients with EoE and pseudodiverticulosis (*n* = 5)	*p* value
*Demographics*				
Age, mean (SD)	50.8 (17.4)	58.1 (15.9)	34.8 (6.4)	0.019^*∗*^
Male sex, *n* (%)	11 (68.8)	7 (63.6)	4 (80.0)	NS
Female sex, *n* (%)	5 (31.2)	4 (36.4)	1 (20.0)	
BMI, mean (SD)	25.1 (4.5)	23.9 (5.1)	27.6 (2.0)	0.221
Smoking, *n* (%)	8 (50.0)	6 (54.5)	1 (20.0)	0.308
Alcohol abuse, *n* (%)	4 (25.0)	4 (36.4)	0 (0)	0.245

*Comorbidities*				
Allergies, including asthma and atopic dermatitis, *n* (%)	7 (43.8)	2 (18.1)	5 (100)	0.005^*∗*^
GERD, *n* (%)	14 (87.5)	11 (100.0)	3 (60.0)	0.083
Diabetes mellitus, *n* (%)	2 (12.5)	2 (18.1)	0 (0)	NS

*Clinical presentation*				
Dysphagia to solids, *n* (%)	14 (87.5)	9 (81.8)	5 (100)	NS
Dysphagia to solids and liquids, *n* (%)	2 (12.5)	2 (18.1)	0 (0)	
Food bolus obstruction, *n* (%)	9 (56.3)	4 (36.4)	5 (100)	0.034^*∗*^

*Endoscopic findings*				
Pseudodiverticulosis of the proximal esophagus, *n* (%)	8 (50.0)	9 (81.8)	0 (0)	0.001^*∗*^
Pseudodiverticulosis of the mid-to-distal esophagus, *n* (%)	6 (37.5)	1 (9.1)	5 (100)	
Diffuse pseudodiverticulosis, *n* (%)	1 (6.3)	1 (9.1)	0 (0)	
Typical endoscopic features of EoE (multiringed esophagus and linear furrows)	5 (31.2)	0 (0)	5 (100)	0.0002
Esophageal Stricture, *n* (%)	11 (68.8)	8 (72.7)	3 (60.0)	NS
*Candidiasis*, *n* (%)	2 (12.5)	2 (18.1)	0 (0)	NS

EIPD: esophageal intramural pseudodiverticulosis; EoE: eosinophilic esophagitis; SD: standard deviation; NS: not significant; BMI: body mass index; GERD: gastroesophageal reflux disease.

^*∗*^Significant differences between groups.

**Table 2:** The clinical course, therapeutic interventions, and outcomes for all patients, including subgroup analysis of patients with EIPD and with EoE and pseudodiverticulosis.

Characteristics	All patients (*n* = 16)	Patients with EIPD (*n* = 11)	Patients with EoE and pseudodiverticulosis (*n* = 5)	*p* value
Total duration of follow-up (months), mean (SD)	44.4 (38.1)	52.2 (42.6)	27.8 (20.0)	0.360

*Endoscopic treatment*				
Dilatation (balloon, bougienage), *n* (%)	9 (56.3)	7 (63.6)	2 (40.0)	0.596
Balloon dilatation, *n* (%)	1 (6.3)	1 (9.1)	0 (0)	NS
Bougienage, *n* (%)	7 (43.8)	6 (54.5)	1 (20.0)	0.308
Total number of endoscopic dilatations, mean (SD)^*∗*^	2.7 (2.5)	1.67 (1.2)	3.0 (2.8)	0.643

*Mortality*				
Deaths, *n* (%)	1 (6.3)	1 (9.1)	0 (0)	NS

EIPD: esophageal intramural pseudodiverticulosis; EoE: eosinophilic esophagitis; SD: standard deviation; NS: not significant.

^*∗*^Mean value calculated only for patients receiving endoscopic dilatations.

## Appendix

See Tables 1 and 2.

## Abbreviations

EoE: Eosinophilic esophagitis
EIPD: Esophageal intramural pseudodiverticulosis.
